# COVID-19 pneumonia in Galicia (Spain): Impact of prognostic factors and therapies on mortality and need for mechanical ventilation

**DOI:** 10.1371/journal.pone.0253465

**Published:** 2021-06-23

**Authors:** Luis Pérez-de-Llano, Eva María Romay-Lema, Adolfo Baloira-Villar, Christian Anchorena, María Luisa Torres-Durán, Adrián Sousa, Dolores Corbacho-Abelaira, José Paz-Ferrin, Carmen Diego-Roza, Laura Vilariño-Maneiro, Pedro J. Marcos, Carmen Montero-Martínez, Fernando de la Iglesia-Martínez, Vanessa Riveiro-Blanco, Nuria Rodríguez-Núñez, José Abal-Arca, María Bustillo-Casado, Rafael Golpe

**Affiliations:** 1 Pneumology Service, Lucus Augusti University Hospital, EOXI Lugo, Monforte, Cervo, Lugo, Spain; 2 Infectious Diseases Unit, Lucus Augusti University Hospital, EOXI Lugo, Monforte, Cervo, Lugo, Spain; 3 Pneumology Service, Complexo Hospitalario Universitario, Pontevedra, Spain; 4 Pneumology Service, Complexo Hospitalario Universitario de Vigo, Vigo, Spain; 5 Internal Medicine Service, Complexo Hospitalario Universitario de Vigo, Vigo, Spain; 6 Pneumology Service, Hospital POVISA, Vigo, Spain; 7 Internal Medicine Service, Hospital POVISA, Vigo, Spain; 8 Pneumology Service, Hospital Arquitecto Marcide, Ferrol, Spain; 9 Internal Medicine Service, Hospital Arquitecto Marcide, Ferrol, Spain; 10 Pneumology Service, Complexo Universitario de A Coruña, A Coruña, Spain; 11 Internal Medicine Service, Complexo Universitario de A Coruña, A Coruña, Spain; 12 Pneumology Service, Complexo Hospitalario Universitario de Santiago, Santiago de Compostela, Spain; 13 Pneumology Service, Complexo Hospitalario Universitario de Ourense, Ourense, Spain; 14 Infectious Diseases Unit, Complexo Hospitalario Universitario de Ourense, Ourense, Spain; University of Sassari, ITALY

## Abstract

**Introduction:**

This study was aimed to identify risk factors associated with unfavorable outcomes (composite outcome variable: mortality and need for mechanical ventilation) in patients hospitalized in Galicia with COVID-19 pneumonia.

**Methods:**

Retrospective, multicenter, observational study carried out in the 8 Galician tertiary hospitals. All Patients admitted with confirmed COVID-19 pneumonia from 1st of March to April 24th, 2020 were included. A multivariable logistic regression analysis was performed in order to identify the relationship between risk factors, therapeutic interventions and the composite outcome variable.

**Results:**

A total of 1292 patients (56.1% male) were included. Two hundred and twenty-five (17.4%) died and 327 (25.3%) reached the main outcome variable. Age [odds ratio (OR) = 1.03 (95% confidence interval (CI): 1.01–1.04)], CRP quartiles 3 and 4 [OR = 2.24 (95% CI: 1.39–3.63)] and [OR = 3.04 (95% CI: 1.88–4.92)], respectively, Charlson index [OR = 1.16 (95%CI: 1.06–1.26)], SaO2 upon admission [OR = 0.93 (95% CI: 0.91–0.95)], hydroxychloroquine prescription [OR = 0.22 (95%CI: 0.12–0.37)], systemic corticosteroids prescription [OR = 1.99 (95%CI: 1.45–2.75)], and tocilizumab prescription [OR = 3.39 (95%CI: 2.15–5.36)], significantly impacted the outcome. Sensitivity analysis using different alternative logistic regression models identified consistently the ratio admissions/hospital beds as a predictor of the outcome [OR = 1.06 (95% CI: 1.02–1.11)].

**Conclusion:**

These findings may help to identify patients at hospital admission with a higher risk of death and may urge healthcare authorities to implement policies aimed at reducing deaths by increasing the availability of hospital beds.

## Introduction

In late 2019, a new coronavirus (now called severe acute respiratory syndrome coronavirus 2: SARS-CoV-2) was identified in a number of patients presenting with pneumonia in Wuhan, Hubei province, China, and since then, virtually all countries have been affected by the pandemic. The severity of coronavirus disease-2019 (COVID-19) ranges from mild symptoms of upper respiratory tract infection to severe pneumonia and, although most reported cases are at the mild end of the spectrum, 15% of infected patients develop viral pneumonia and 1–5% of them die [1]. The fatality rate differs considerably across countries and different regions of the same country, most likely reflecting overwhelming of healthcare systems [2].

Over the past months, we have learnt what risk factors are most likely to impact on the prognosis [3–6] but it remains to be clarified how the different therapeutic strategies (antivirals, systemic corticosteroids, hydroxychloroquine, tocilizumab, antibiotics) may influence the outcome of the patients [7–9].

The aim of this study was to identify clinical, demographic and therapeutic risk factors associated with worse outcomes (mortality and need for mechanical ventilation) in patients hospitalized in Galicia with COVID-19 pneumonia during the spring 2020 outbreak.

## Material and methods

### Design

Retrospective, multicenter, observational study carried out in the 8 Galician tertiary hospitals (Complexo Hospitalario Universitario de A Coruña, Hospital Universitario Lucus Augusti, Complexo Hospitalario Universitario de Ferrol, Complexo Hospitalario Universitario de Ourense, Complexo Hospitalario Universitario de Vigo, Hospital Policlínico de Vigo, Complexo Hospitalario Universitario de Pontevedra, Complexo Hospitalario Universitario de Santiago). These 8 hospitals cover about 85% of the Galician population. Galicia has a population of 2,699,449 inhabitants (1,298,964 male), with a mean age of 47.2 y (44 y in Spain). It is an ageing population (515,847 over 70), particularly vulnerable to severe COVID-19 infection.

All Patients admitted with confirmed COVID-19 pneumonia from 1st of March to April 24th, 2020 were included. Confirmed COVID-19 infection was defined as: “Clinical criteria (at least, 2 of the following symptoms: fever, cough, headache, myalgias, diarrhea, asthenia) plus detection of SARS-CoV-2 from a clinical specimen using a validated PCR or significant rise of IgG antibody level to SARS-CoV-2 between paired sera plus radiologic infiltrates”.

### Compliance with ethical standards

The study was approved by the ethics committee of Galicia (Cod. 2020/239). Data were de-identified for analysis. Informed consent was waived due to the retrospective, non-interventional design of the study and the use of anonymous clinical data for the analysis.

### Data collection

Galicia has a network of 8 tertiary hospitals interconnected with a shared operating system that provides a unified electronic medical record, giving the researchers the opportunity to collect good quality data. Data were processed internally and in pseudonymised form were collected by two independent data managers from the electronic medical records. Demographic data, risk factors for poor outcome (cardiovascular diseases, arterial hypertension, diabetes, chronic bronchial diseases, renal failure, cancer, hepatic failure, Kidney transplantation, other organ transplantation), laboratory data on admission (D dimers, lymphocyte and platelet counts, PaO2, VSG, PCR…) and COVID-19 treatments used during admission (antiretroviral drugs, anti-malarial drugs, monoclonal antibodies, systemic corticosteroids, interferon, antibiotics) were collected.

### Statistical analysis

Main outcome variable was a composite of in-hospital death (every death in patients included in this study was allocated to COVID-19 pneumoniae irrespective of secondary complications), and need for mechanical ventilation. Secondary outcome variable was in-hospital death. Category variables are presented in terms of absolute and relative frequencies, median and interquartile range. Normality was evaluated using the Kolmogorov-Smirnov test with Lilliefords´ correction. Between-groups comparisons were made by means of Chi-squared test for categorical variables and either Student’s t-test or Mann-Whitney U test for continuous variables, as appropriate.

A multivariable logistic regression analysis was performed in order to identify the relationship between risk factors, therapeutic interventions and the composite results variable. Variables with biological plausibility were entered simultaneously in one single step, without checking. We used the following independent variables: age, sex, crude (non-age-adjusted) Charlson comorbidity index, C-reactive protein (CRP), arterial oxygen saturation (SaO_2_) and the different treatments used during admission. We also included a ratio ([number of admissions per hospital/number of hospital beds] x 100) as a measure of the hospitals’ bed stress. Age, Charlson index, SaO_2_ and the ratio of hospitals’ bed stress were coded continuously. Sex and treatments were coded dichotomously. CRP was selected as a marker of inflammatory status because it was available for most patients. Because not all hospitals used the same measuring method and reference values for CRP, this variable was coded in each hospital in quartiles.

We performed a sensitivity analysis, using several different models. In these alternative analyses we replaced CRP in the multivariable analysis by the lymphocyte and neutrophils cell count, and also added the platelet count and the procalcitonin values. We used both a method that entered all the variables in the model without checking, and a forward automatic conditional selection method that sequentially entered variables with a p-value < 0.05.

Statistical tests were two-sided and significance was taken at the level of p < 0.05. We used MedCalc Statistical Software version 13.3.3 (MedCalc Software bvba, Ostend, Belgium). Strobe checklists can be found in S1 Appendix.

## Results

### Demographic characteristics

A total of 1292 patients (56.0% male) were included in this study. Mean age was 68.8 ± 14.5 years [median: 70.7 (interquartile range [IQR]: 59.5–79.3)]. Table 1 shows demographic characteristics of the sample and the prevalence of the main pre-existing comorbidities. The most frequent ones were arterial hypertension (49.7%), diabetes mellitus (20.2%), ischemic heart disease (12.6%) and atrial fibrillation (12.5%).

**Table 1:** Demographic characteristics and comorbidity data of study subjects.

Variable	Total n = 1292	Neither death, nor MV n = 965	Death or MV n = 327	*P* value
Male, frequency (%)	724 (56.0)	520 (53.8)	204 (62.3)	0.007
Age, median (IQR)	70.7 (59.7–79.4)	68.2 (56.9–76.0)	76.1 (68.2–85.0)	< 0.001
< 40 yrs, frequency (%)	46 (3.6)	43 (4.4)	3 (0.9)	< 0.0001
[40–49) yrs frequency (%)	110 (8.5)	100 (10.3)	10 (3.0)	
[50–59) yrs frequency (%)	172 (13.3)	148 (15.3)	24 (7.3)	
[60–69) yrs frequency (%)	297 (23.0)	241 (24.9)	56 (17.1)	
[70–79) yrs frequency (%)	358 (27.7)	255 (26.4)	103 (31.4)	
≥80 yrs frequency (%)	309 (23.9)	178 (18.4)	131 (40.0)	
Arterial hypertension frequency (%)	642 (49.7)	442 (45.8)	200 (61.1)	<0.001
Diabetes mellitus frequency (%)	261 (20.2)	164 (16.9)	97 (29.6)	<0.001
Heart failure frequency (%)	99 (7.7)	54 (5.5)	45 (13.7)	<0.001
Ischemic heart disease frequency (%)	163 (12.6)	98 (10.1)	65 (19.8)	<0.001
Atrial fibrillation frequency (%)	161 (12.5)	108 (11.1)	53 (16.2)	0.018
Peripheral arterial vasculopathy frequency (%)	117 (9.1)	67 (6.9)	50 (15.2)	<0.001
Asthma frequency (%)	93 (7.2)	76 (7.8)	17 (5.1)	0.10
COPD frequency (%)	67 (5.2)	43 (4.4)	24 (7.3)	0.04
Bronchiectasis frequency (%)	39 (3.0)	19 (1.9)	20 (6.1)	<0.001
Interstitial lung disease frequency (%)	8 (0.6)	5 (0.5)	3 (0.9)	0.42
Chronic kidney disease frequency (%)	100 (7.7)	52 (5.3)	48 (14.6)	<0.001
Dementia frequency (%)	117 (9.1)	60 (6.2)	57 (17.4)	<0.001
Chronic liver disease frequency (%)	47 (3.6)	35 (3.6)	12 (3.6)	0.97
Cancer frequency (%)	106 (8.2)	64 (6.6)	42 (12.8)	<0.001
Leukemia or lymphoma frequency (%)	29 (2.2)	22 (2.2)	7 (2.1)	0.91

MV: mechanical ventilation. IQR: interquartile range. COPD: chronic obstructive pulmonary disease.

### Clinical and laboratory features. Therapy prescribed on admission

Mean days from symptom onset to admission was 7.5 ± 5.1 (median 7.0, IQR: 4.0–10.0). The most common symptoms were (S1 Table): fever (77.6%), cough (74.5%) and dyspnea (53.3%). Laboratory findings at admission are shown in Table 2. Of note, mean PaO_2_ was 63.9 ± 15.6 mmHg [median: 64 (IQR: 55–74)] and mean SaO_2_ was 92.1 ± 8.1% [median: 94 (IQR: 92–96)]. Two hundred and sixteen (16.7%) patients presented with respiratory failure.

**Table 2:** Laboratory findings on admission.

Variable	Total n = 1292	Neither death, nor MV n = 965	Death or MV n = 327	*P* value
**Haemoglobin**				
No. of patients with data	1288			
Median (IQR) g/L	13.6 (12.5–14.6)	13.6 (12.6–14.6)	13.4 (11.9–14.6)	0.009
**WBC count**				
No. of patients with data	1289			
Median (IQR) (cells x 10^3^/mm3)	5.6 (4.3–7.7)	5.4 (4.3–7.3)	6.3 (4.8–9.2)	< 0.0001
**Neutrophil count**				
No. of patients with data	1163			
Median (IQR) (cells x 10^3^/mm3)	4.1 (2.9–6.0)	3.8 (2.8–5.6)	5.1 (3.5–7.9)	< 0.0001
**Lymphocyte count**				
No. of patients with data	1281			
Median (IQR) (cells x 10^3^ /mm3)	0.9 (0.6–1.2)	0.9 (0.7–1.3)	0.7 (0.5–1.0)	< 0.0001
**Platelets**				
No. of patients with data	1282			
Median (IQR) (cells x 10^3^/mm3)	176.0 (137.0–230.0)	178.0 (138.0–235.0)	171.5 (132.0–221.0)	0.03
**D-dimer**				
No. of patients with data	980			
Median (IQR) (ng/ml)	710.5 (440.5–1225.5)	644.0 (418.– 1126.0)	931.0 (634–1710.0)	< 0.0001
**CRP**				
No. of patients with data	1255			< 0.0001
1^st^ quartile	317 (25.3)	275 (29.2)	42 (13.4)	
2^nd^ quartile	311 (24.8)	245 (26.0)	66 (21.0)	
3^rd^ quartile	313 (24.9)	238 (25.3)	75 (24.0)	
4^th^ quartile	314 (25.0)	183 (19.4)	131 (41.7)	
**Procalcitonin**				
No. of patients with data	1036			
Median (IQR) (μg/L)	0.1 (0.06–0.2)	0.09 (0.05–0.14)	0.20 (0.10–0.50)	< 0.0001
**Creatinine**				
No. of patients with data	1284			
Median (IQR) (mg/dl)	0.89 (0.80–1.10)	0.83 (0.80–1.02)	1.04 (0.81–1.43)	< 0.0001
**ALT**				
No. of patients with data	917			
Median (IQR) (U/L)	36.0 (27.0–54.0)	34.0 (26.0–49.0)	46.0 (32.0–68.0)	< 0.0001
**Lactate dehydrogenase**				
No. of patients with data	917			
Median (IQR) (U/L)	353.0 (250.0–521.0)	324.0 (237.2–478.5)	443.5 (317.5–672.5)	< 0.0001
**Serum albumin**				
No. of patients with data	637			
Median (IQR) (g/dL)	3.7 (3.3–3.9)	3.7 (3.3–3.9)	3.5 (3.1–3.8)	0.0001
**Troponin I**				
No. of patients with data	429			
Median (IQR) (ng/L)	16.0 (10.0–22.0)	16.0 (9.0–16.0)	20.0 (16.0–87.0)	< 0.0001
**Ferritin**				
No. of patients with data	582			
Median (IQR) (μg/L)	481.3 (237.0–1007.0)	450.0 (209.5–921.5)	583. 0 (291.0–1304.0)	0.007
**Interleukin 6**				
No. of patients with data	291			
Median (IQR) (pg/mL)	29.8 (14.2–51.79	27.2 (11.2–43.3)	50.8 (22.5–107.5)	< 0.0001
**PaO2**				
No. of patients with data	829			
Median (IQR) (mmHg)	64.0 (55.0–74.0)	67.0 (59.0–72.2)	56.0 (47.0–68.0)	< 0.0001
**SaO2 (%)**				
No. of patients with data	1270			
Median (IQR)	94.0 (92.0–96.0)	95.0 (93.0–97.0)	92.0 (85.0–94.0)	< 0.0001

MV: mechanical ventilation. IQR: interquartile range. WBC: white blood cell. CRP: C-reactive protein. ALT: alanine transaminase.

Lopinavir/ritonavir was administered to 62.5% of patients, whereas 37.1% received systemic corticosteroids and tocilizumab was prescribed in 9.4% patients. Hydroxychloroquine (400 mg twice daily the first day followed by 200 mg twice daily for five days) and azithromycin (500 mg daily for 5 days) were employed in 91.9% and 79.2% of the patients, respectively (Table 3). It deserves to be emphasized that the patients who received systemic corticosteroids and tocilizumab had a more severe clinical condition on admission, whilst the ones who received hydroxychloroquine presented with a milder clinical picture (S2 Table).

**Table 3:** Medications administered during hospitalization and prior to mechanical ventilation (MV).

Variable	Total (n = 1292)	Neither death, nor MV (n = 965)	Death or MV (n = 327)	*P* value
Hydroxychloroquine, n (%)	1187 (91.9)	924 (98.8)	263 (80.4)	<0.001
Azithromycin, n (%)	1023 (79.2)	761 (78.8)	262 (80.1)	0.62
Empiric antibiotics, n (%)	854 (66.1)	626 (64.8)	228 (69.7)	0.10
Lopinavir/ritonavir, n (%)	807 (62.5)	615 (63.7)	192 (58.7)	0.10
Systemic corticosteroids, n (%)	479 (37.1)	297 (30.7)	182 (55.6)	<0.001
Maximum daily dose of corticosteroids*, mean (SD)	67.4 (46.5)	66.3 (44.8)	69.1 (49.3)	0.55
Tocilizumab, n (%)	121 (9.4)	61 (6.3)	60 (18.3)	<0.001

*Calculated as prednisone-equivalent dose.

### Outcome results

Of the total 1292 patients, 225 (17.4%) died, 149 (11.5%) were transferred to the Intensive care Unit and received mechanical ventilation and 327 (25.3%) either died or needed mechanical ventilation (main composite outcome variable). Median time from admission to mechanical ventilation was 2.0 days (IQR, 1.0–4.0) and the median of the hospital stay was 10 days (IQR, 6.0–15.0).

### Risk factors for mortality or need for mechanical ventilation

The predictors of mortality or need for mechanical ventilation in the multivariable analysis were as follows (Table 4): age [odds ratio (OR) = 1.03 (95% confidence interval (CI): 1.01–1.04)], CRP quartiles 3 and 4 [OR = 2.24 (95% CI: 1.39–3.63)] and [OR = 3.04 (95% CI: 1.88–4.92)], respectively, Charlson index [OR = 1.16 (95%CI: 1.06–1.26)], SaO_2_ upon admission [OR = 0.93 (95% CI: 0.91–0.95)], hydroxychloroquine prescription [OR = 0.22 (95%CI: 0.12–0.37)], systemic corticosteroids prescription [OR = 1.99 (95%CI: 1.45–2.75)], and tocilizumab prescription [OR = 3.39 (95%CI: 2.15–5.36)].

**Table 4:** Results of the multivariable logistic regression analysis for death or need of mechanical ventilation.

Variable	Odds ratio	95% CI	P
Age	1.03	1.01 to 1.04	<0.001
Female sex	0.82	0.59 to 1.13	0.23
Charlson index	1.16	1.06 to 1.26	0.001
CRP quartile 2*	1.64	0.99 to 2.70	0.051
CRP quartile 3*	2.24	1.39 to 3.63	0.001
CRP quartile 4*	3.04	1.88 to 4.92	<0.001
SaO_2_	0.93	0.91 to 0.95	<0.001
Corticosteroids	1.99	1.45 to 2.75	<0.001
Tocilizumab	3.39	2.15 to 5.36	<0.001
Hydroxychloroquine	0.22	0.12 to 0.37	<0.001
Empiric antibiotics	1.06	0.72 to 1.57	0.73
Azithromycin	0.82	0.51 to 1.31	0.40
Lopinavir-ritonavir	1.14	0.81 to 1.62	0.43
Ratio admissions/hospital beds	1.04	0.99 to 1.08	0.06

*Quartile 1 is the reference. For definitions, see legend to Table 2.

We carried out an additional analysis using in-hospital mortality as a secondary outcome variable. Results are shown in Table 5. The association between corticosteroids treatment and an increased risk of death did not reach statistical significance, while there were significant associations between this outcome and age, comorbidity burden, CRP, SaO_2_, hydroxychloroquine and tocilizumab, similar to those found for the main composite outcome variable.

**Table 5:** Results of the multivariable logistic regression analysis for in-hospital death.

Variable	Odds ratio	95% CI	P
Age	1.07	1.05 to 1.09	<0.001
Female sex	0.71	0.48 to 1.06	0.09
Charlson index	1.30	1.18 to 1.44	<0.001
CRP quartile 2*	2.04	1.06 to 3.91	0.03
CRP quartile 3*	3.11	1.68 to 5.75	<0.001
CRP quartile 4*	3.39	1.82 to 6.28	<0.001
SaO_2_	0.94	0.92 to 0.95	<0.001
Corticosteroids	1.44	0.97 to 2.11	0.06
Tocilizumab	1.95	1.10 to 3.45	0.02
Hydroxychloroquine	0.27	0.15 to 0.48	<0.001
Empiric antibiotics	0.79	0.49 to 1.25	0.31
Azithromycin	0.64	0.37 to 1.10	0.10
Lopinavir-ritonavir	0.89	0.59 to 1.34	0.58
Ratio admissions/hospital beds	0.97	0.92 to 1.02	0.32

*Quartile 1 is the reference. For definitions, see legend to Table 2.

The results of the sensitivity analysis for the main outcome variable are shown in S3 and S4 Tables. The results of the analysis did not significantly change in these additional analyses, except for the fact that the ratio admissions/hospital beds consistently and significantly correlated with the outcome [OR: 1.69 (95% CI: 1.02–1.18)]

## Discussion

### Mortality and comparison with other series

We found a 17.2% in-hospital mortality in this cohort, formed by patients admitted with COVID-19 pneumonia in 8 tertiary Galician hospitals during the peak of the outbreak in March and April 2020. Among 126,137 patients hospitalized for COVID-19 from March to July 2020 in the USA, 15% of patients (19,594) died during the index hospitalization [10]. Du et al reported a 11.7% fatality rate among 179 hospitalized COVID-19 patients [11]. These rates are lower than the one we observed, but it is worth to mention that not all patients included in those studies were diagnosed as having pneumonia. Mortality figures were also higher in our series than in the Chen´s one (11.1%), but although this cohort was entirely formed by pneumonia-diagnosed patients, it should be remarked that mean age was much lower (55 yrs vs 68 yrs) and PaO2 was higher (72.0 vs 63.9 mmHg), likely reflecting less severe condition at admission [12]. In Spain, Berenguer et al found a 28% mortality rate in 4,035 hospitalized patients with COVID-19 disease, of which 77% presented with infiltrates on chest radiograph [13]. Rubio-Rivas et al reported a mortality that exceeded 15% among 12,066 patients admitted with COIVD-19 infection (the rate of those diagnosed with pneumonia has not been specified) in 109 Spanish hospitals [14]. More recently published clinical trials reported mortality rates between 5.1 and 10.4%, but included patients were recruited as late as July 2020 and mean age was considerably lower (55 years) [15, 16].

Data on COVID-19 pneumonia mortality during the second wave are lacking and the scarce available information suggests that mortality rates have declined, at least in developed countries, but this observation could be explained by the fact that second-wave infections tended to affect younger people and it cannot be attributed with certainty to therapeutic advances [17–19]. Another plausible explanation is provided by Asch and colleagues, who found that the risk-adjusted mortality decreased from 16.56% to 9.29% in the early period of their study (January through April 2020) compared with the later period (May through June 2020), reflecting a possible association between an increased in-hospital mortality and a high prevalence of COVID-19 in the community [20].

### Risk factors for unfavorable outcome

We identified predictors for poor outcome (need for mechanical ventilation or in-hospital death) in patients admitted with Covid-19 pneumonia: old age, low SaO_2_, high CRP values, preexistence of comorbidities and the use of systemic corticosteroids and tocilizumab. Hydroxychloroquine prescription was associated with a favorable outcome.

Increased age has been repeatedly associated with adverse outcome [21, 22] and case fatality rate was reported to be higher in patients with comorbidities, in particular cardiovascular diseases and diabetes [23–26]. On the other hand, low blood oxygen saturation has also been used to identify severe COVID-19 pneumonia in admitted patients [27] and admission CRP correlated with disease severity and tended to be a good predictor of adverse outcome [28, 29].

In this study we have found that anti-inflammatory drugs (systemic corticosteroids and tocilizumab) negatively impact on prognosis, even after adjusting for other potential risk factors. In the earlier period of the pandemic, some influential experts advised not to use systemic corticosteroids in patients with COVID-19 pneumonia, relying on the lack of evidence, the negative results observed in influenza pneumonia and impaired clearance of SARS-CoV [30]. Ever since, several studies with an observational design (either retrospective or prospective) yielded contradictory results, with some reporting a positive impact on survival [31–37], while others concluding the opposite [38, 39]. The only prospective and randomized clinical trial (although not double-blinded), demonstrated that dexamethasone treatment reduced deaths by one-third in mechanically ventilated patients and by one-fifth in patients receiving oxygen only, whereas no difference in mortality was found in patients who did not need any breathing support [40]. The results of three meta-analysis further support the employ of systemic corticosteroids in COVID-19 pneumonia, but it is worth to mention that the weight of RECOVERY trial was more than 50 per cent in all of them and the magnitude of the effect was modest (OR ranging from 0.70 to 0.88) [41–43]. On the other hand, several propensity score matching (PSM) studies found no impact of corticosteroids on COVID-19 pneumonia outcome [44–49], while an increased mortality was observed in other report [50] and two additional PSM studies concluded that corticosteroid therapy was associated with lower mortality [51, 52]. Therefore, considering all the available evidence, the role of corticosteroids remains controversial in non-critically ill patients who need supplemental oxygen. The evidence for the use of tocilizumab is far less, since only non-randomized single-arm studies and case-series were published with promising results [36, 53–55]. Recently published studies seem to indicate that tocilizumab has additional benefit to corticosteroids in patients with clinically progressive disease [56, 57], whereas this treatment did not result in significantly lower mortality than placebo when given as monotherapy in most of the included patients [58].

In contrast, we have found a protective effect of hydroxychloroquine on death or need for mechanical ventilation, whilst investigators of the Recovery Trial reported that results convincingly exclude any significant mortality advantage of hydroxychloroquine in hospitalized COVID-19 patients [59].

Therefore, our results seem to be counterintuitive and two explanations can be put forward to account for these differences. The first one is obvious: in our population, corticosteroids and tocilizumab worsen the prognosis of patients with COVID-19 pneumonia. In fact, we have performed an exhaustive regression analysis containing significant covariates in an attempt to simultaneously adjust for their effect with the hope of isolating the impact of the intervention. The second explanation has to do with the inherent drawback of retrospective studies: the existence of potential bias and/or confounders. It is important to note that the captured data reflect the patients´ clinical condition at the moment of admission, but this situation changes over time, with some patients improving and some deteriorating, and this surely influences the physician´s decision to prescribe or not a given drug. Since this study was not performed under a controlled condition and treatments were not randomly assigned, we acknowledge that confounding by indication may still persist. Indeed, anti-inflammatory treatment was prescribed in patients with a more severe clinical condition and statistical adjustment for known confounders may not suffice to arrive at 2 groups that have truly the same prognosis at baseline (or, maybe more importantly, in the precise moment that the drug was administered). With hydroxychloroquine it happened exactly the opposite: it was given to patients with less severe disease.

Interestingly, we found that hospital stress was associated with a higher risk of unfavorable outcomes in sensitive analysis, applying different regression schemes (S3 and S4 Tables). A total of 178 out of the 225 deceased patients did not receive mechanical ventilation, but the decision making was not influenced by a lack of ICU resources and clinical criteria based on the likelihood of survival were applied in every case. Although we are not aware of prior studies reporting the association between hospital burden and mortality in patients with COVID-19, this is plausible because hospitals perform worse when they are overwhelmed. Nonetheless, it has been published that the quality of public health systems might be decisive in determining health benefits against pandemics [60]. Moreover, death rate in the North of Italy was twice as high as in the Center-South (13.03% and 6.65%, respectively) during the earlier phase of the current COVID-19 pandemic, likely reflecting a lower hospital stress in the latter (0.81 and 0.20, respectively) [61].

### Strengths and limitations

The study’s strengths include the large sample size and the homogeneity of the included population (all patients had microbiologically proven COVID-19 infection and were radiologically diagnosed with pneumonia). Besides, the fact that all Galician hospitals share the same model of electronic medical record enabled us to include every patient admitted to hospital during the study period. Baseline demographic, clinical and laboratory characteristics, alongside the presence of significant comorbidities were individually and exhaustively studied. We also evaluated the effectiveness of viral and host-targeted medications. The main limitation is its retrospective character leading to missing data. Moreover, as aforementioned, studies with retrospective design are not the most accurate to evaluate the impact of treatment on prognosis and survival, due to the possible presence of unnoticed and uncontrolled confounders. Another limitation lies in the unavailability of certain variables that would have been of interest for the aim of our study, such as body mass index.

## Conclusion

In conclusion, we identified several predictors of mortality (age, comorbidities, CRP and SpO2 values, the hospitals’ burden, the use of corticosteroids and tociluzumab, and the non-use of hydroxychloroquine) among a large cohort of COVID-19 pneumonia patients consecutively admitted to hospitals in Galicia (Spain) during the first outbreak of the epidemic. These findings offer a valuable insight into the characteristics and outcome of patients hospitalized with COVID-19 pneumonia. Our results also suggest that countries have the opportunity to implement policies aimed at reducing deaths by increasing the availability of hospital beds. Results concerning the efficacy of treatments should be interpreted with caution and more well-designed prospective studies are needed to clarify the role of the different therapies in this clinical context.

## Supporting information

S1 Appendix STROBE statement—checklist of items that should be included in reports of observational studies. (DOCX)

S1 Table Symptoms and signs on admission. (DOCX)

S2 Table Differences between patients who received or not corticosteroids, tocilizumab and hydroxychloroquine. (DOCX)

S3 Table Results of the sensitivity analysis, replacing CRP values by lymphocyte and neutrophil count. Model 1: enter variables method; Model 2: forward automatic selection method.
(DOCX)

S4 Table Results of the sensitivity analysis, replacing CRP values by lymphocyte and neutrophil count and including procalcitonin and platelet count. Model 1: enter variables method; Model 2: forward automatic selection method.
(DOCX)
