# Supplementary material for: COVID-19 pneumonia in Galicia (Spain): Impact of prognostic factors and therapies on mortality and need for mechanical ventilation
Source: PLoS One. 2021 Jun 23;16(6):e0253465. doi: 10.1371/journal.pone.0253465 (PMC8221482; doi:10.1371/journal.pone.0253465)
Supplement: S1 Table — (DOCX) [file pone.0253465.s002.docx]

**S1 Table. Symptoms and signs on admission.**

| **Variable** | **Values** |
| --- | --- |
| Days from symptoms onset | 7.0 (4.0 – 10.0) |
| Fever | 1002 (77.6) |
| Cough | 963 (74.5) |
| Dyspnea | 689 (53.3) |
| Myalgia | 328 (25.4) |
| Diarrhea | 233 (18.0) |
| Cephalea | 112 (8.7) |
| Chest pain | 110 (8.5) |
| Nausea | 94 (7.3) |
| Vomiting | 79 (6.1) |
| Confusion | 66 (5.1) |
| Hemoptysis | 15 (1.2) |

Data are expressed as median (IQR) or as n (%)
