# Supplementary material for: COVID-19 pneumonia in Galicia (Spain): Impact of prognostic factors and therapies on mortality and need for mechanical ventilation
Source: PLoS One. 2021 Jun 23;16(6):e0253465. doi: 10.1371/journal.pone.0253465 (PMC8221482; doi:10.1371/journal.pone.0253465)
Supplement: S2 Table — (DOCX) [file pone.0253465.s003.docx]

**S2 Table. Differences between patients who received or not corticosteroids, tocilizumab and hydroxychloroquine.**

|  | Corticosteroids | | | Tocilizumab | | | Hidroxicloroquine | | |
| --- | --- | --- | --- | --- | --- | --- | --- | --- | --- |
|  | Yes (N=479) | No (N=813) | P | Yes (N=121) | No (N=1171) | P | Yes (N=1187) | No (N=105) | p |
| Age | 73.1 (63.2 – 82.4) | 68.6 (56.5 – 78.5) | <0.0001 | 69.7 (62.6 – 76.4) | 70.8 (58.7 – 80.7) | 0.58 | 69.9 (59.1 – 78.2) | 83.7 (65.3 – 88.8) | < 0.0001 |
| Sex (male) | 294 (61.4) | 431 (53.0) | 0.004 | 84 (69.4) | 641 (54.7) | 0.002 | 670 (56.4) | 55 (52.4) | 0.48 |
| Charlson index | 1 (0 – 2) | 0 (0 – 2) | < 0.0001 | 1 (0 -2) | 1 (0 – 2) | 0.82 | 0 (0 – 2) | 2 (0 -3) | < 0.0001 |
| SaO_2_ | 93 (89 – 96) | 95 (93 – 97) | < 0.0001 | 93 (90 – 95) | 95 (92 – 97) | < 0.0001 | 95 (92 – 96) | 92 (85 – 96) | 0.0004 |
| PCR |  |  | < 0.0001 |  |  | 0.0001 |  |  | 0.006 |
| Quartile 1 | 86 (18.5) | 235 (29.7) |  | 12 (10.0) | 309 (27.2) |  | 300 (26.0) | 21 (21.0) |  |
| Quartile 2 | 122 (26.3) | 190 (24.0) |  | 36 (30.0) | 276 (24.3) |  | 295 (25.5) | 17 (17.0) |  |
| Quartile 3 | 110 (23.7) | 199 (25.2) |  | 28 (23.3) | 281 (24.8) |  | 286 (24.8) | 23 (23.0) |  |
| Quartile 4 | 146 (31.5) | 167 (21.1) |  | 44 (36.7) | 269 (23.7) |  | 274 (23.7) | 39 (39.0) |  |

Results are expressed as median (IQR) or as number of observations (%)
