# Supplementary material for: COVID-19 pneumonia in Galicia (Spain): Impact of prognostic factors and therapies on mortality and need for mechanical ventilation
Source: PLoS One. 2021 Jun 23;16(6):e0253465. doi: 10.1371/journal.pone.0253465 (PMC8221482; doi:10.1371/journal.pone.0253465)
Supplement: S3 Table — Model 1: enter variables method; Model 2: forward automatic selection method. (DOCX) [file pone.0253465.s004.docx]

**S3 Table. Results of the sensitivity analysis, replacing CRP** **values by lymphocyte and neutrophil count****.** Model 1: enter variables method; Model 2: forward automatic selection method.

| **Model 1** | | | | **Model 2** | | | |
| --- | --- | --- | --- | --- | --- | --- | --- |
| **Variable** | **Odds ratio** | **95% CI** | **P** | **Variable** | **Odds ratio** | **95% CI** | **P** |
| Age | 1.03 | 1.01-1.04 | <0.001 | Age | 1.02 | 1.01-1.04 | < 0.001 |
| Female sex | 0.83 | 0.60-1.16 | 0.28 | Charlson index | 1.15 | 1.06-1.26 | 0.001 |
| Charlson index | 1.15 | 1.05-1.25 | 0.001 | SaO_2_ | 0.93 | 0.91-0.95 | < 0.001 |
| SaO_2_ | 0.93 | 0.91-0.94 | <0.001 | Corticosteroids | 2.27 | 1.63-3.14 | < 0.001 |
| Corticosteroids | 2.26 | 1.62-3.15 | <0.001 | Tocilizumab | 3.40 | 2.16-5.37 | < 0.001 |
| Tocilizumab | 3.33 | 2.10-5.29 | <0.001 | Hydroxychloroquine | 0.27 | 0.16-0.46 | < 0.001 |
| Hydroxychloroquine | 0.26 | 0.15-0.46 | <0.001 | Lymphocite count | 0.46 | 0.33-0.64 | < 0.001 |
| Empiric antibiotics | 1.10 | 0.73-1.66 | 0.62 | Neutrophil count | 1.10 | 1.05-1.15 | < 0.001 |
| Azithromycin | 0.83 | 0.51-1.34 | 0.46 | Ratio admissions/hospital beds | 1.06 | 1.02-1.11 | 0.004 |
| Lopinavir-ritonavir | 1.09 | 0.75-1.56 | 0.64 |  |  |  |  |
| Lymphocite count | 0.47 | 0.34-0.66 | <0.001 |  |  |  |  |
| Neutrophil count | 1.10 | 1.05-1.14 | <0.001 |  |  |  |  |
| Ratio admissions/hospital beds | 1.06 | 1.02-1.11 | 0.004 |  |  |  |  |
