# Supplementary material for: COVID-19 pneumonia in Galicia (Spain): Impact of prognostic factors and therapies on mortality and need for mechanical ventilation
Source: PLoS One. 2021 Jun 23;16(6):e0253465. doi: 10.1371/journal.pone.0253465 (PMC8221482; doi:10.1371/journal.pone.0253465)
Supplement: S4 Table — Model 1: enter variables method; Model 2: forward automatic selection method. (DOCX) [file pone.0253465.s005.docx]

**S4 Table. Results of the sensitivity analysis, replacing CRP values values by lymphocyte and neutrophil count and including procalcitonin and platelet count**. Model 1: enter variables method; Model 2: forward automatic selection method.

| **Model 1** | | | | **Model 2** | | | |
| --- | --- | --- | --- | --- | --- | --- | --- |
| **Variable** | **Odds ratio** | **95% CI** | **P** | **Variable** | **Odds ratio** | **95% CI** | **P** |
| Age | 1.02 | 1.00-1.03 | 0.006 | Age | 1.02 | 1.00-1.03 | 0.005 |
| Female sex | 1.11 | 0.75-1.64 | 0.5 | Charlson index | 1.16 | 1.05-1.29 | 0.003 |
| Charlson index | 1.17 | 1.06-1.31 | 0.002 | SaO_2_ | 0.90 | 0.87-0.92 | < 0.001 |
| SaO_2_ | 0.90 | 0.87-0.92 | <0.001 | Corticosteroids | 2.17 | 1.47-3.20 | < 0.001 |
| Corticosteroids | 2.13 | 1.44-3.16 | <0.001 | Tocilizumab | 4.08 | 2.29-7.26 | < 0.001 |
| Tocilizumab | 4.09 | 2.27-7.35 | <0.001 | Hydroxychloroquine | 0.18 | 0.09-0.34 | < 0.001 |
| Hydroxychloroquine | 0.17 | 0.09-0.35 | <0.001 | Lymphocite count | 0.52 | 0.35-0.77 | 0.001 |
| Empiric antibiotics | 1.32 | 0.82-2.14 | 0.24 | Neutrophil count | 1.14 | 1.08-1.21 | < 0.001 |
| Azithromycin | 0.79 | 0.45-1.36 | 0.39 | Platelet count | 0.99 | 0.99-0.99 | < 0.001 |
| Lopinavir-ritonavir | 1.10 | 0.72-1.68 | 0.63 | Ratio admissions/hospital beds | 1.05 | 1.01-1.11 | 0.01 |
| Lymphocite count | 0.51 | 0.34-0.77 | 0.001 |  |  |  |  |
| Neutrophil count | 1.14 | 1.03-1.21 | <0.001 |  |  |  |  |
| Platelet count | 0.99 | 0.99-0.99 | <0.001 |  |  |  |  |
| Procalcitonin | 1.01 | 0.93-1.11 | 0.68 |  |  |  |  |
| Ratio admissions/hospital beds | 1.05 | 1.00-1.11 | 0.02 |  |  |  |  |
